# Adult-type granulosa cell tumor with pleural effusion: A rare case report

**DOI:** 10.1097/MD.0000000000042058

**Published:** 2025-03-28

**Authors:** Minghan Chai, Qiuli Jin, Jie Zhang, Ying Zhao, Qing Xue

**Affiliations:** a Department of Gynecology, Shanxian Central Hospital, Heze, Shandong, China.

**Keywords:** granulosa cell tumor, pelvic mass, pleural effusion, postmenopausal women

## Abstract

**Rationale:**

Adult granulosa cell tumors (AGCTs), representing the most prevalent subtype of sex cord-stromal tumors in the ovary, predominantly manifest in perimenopausal and postmenopausal women. A notably uncommon clinical manifestation of AGCTs is the concomitant development of pleural effusion, a condition that may be erroneously attributed to more prevalent etiologies such as cardiac insufficiency, pulmonary infections, or other malignant processes. Consequently, the occurrence of pleural effusion in association with AGCTs is atypical and warrants heightened clinical vigilance for such rare presentations. This article presents a detailed case report, aiming to enhance the timely identification and management of this condition, thereby optimizing patient prognosis.

**Patient concerns:**

A 60-year-old postmenopausal woman presented with a 1-month history of persistent chest tightness and dyspnea. Initial chest radiography demonstrated a right-sided pleural effusion. Thoracentesis was performed, providing transient symptomatic relief; however, the effusion persisted without complete resolution. Further diagnostic evaluation with computed tomography imaging revealed the presence of a pelvic mass, which necessitated surgical intervention for definitive management.

**Diagnoses:**

Histopathological analysis of the surgical specimen confirmed the diagnosis of an adult granulosa cell tumor originating from the left ovary.

**Interventions:**

Postoperative evaluation demonstrated a marked reduction in pleural effusion, with complete resolution achieved during the recovery period.

**Outcomes:**

Throughout the follow-up surveillance, no evidence of tumor recurrence has been detected.

**Lessons:**

This case highlights the pivotal role of multidisciplinary collaboration in addressing complex clinical scenarios. Furthermore, it emphasizes the imperative for early detection and prompt intervention in cases of pelvic masses among postmenopausal women, underscoring the potential for improved clinical outcomes through timely and coordinated management.

## 1. Introduction

Pelvic masses in postmenopausal women, although relatively uncommon, may indicate an underlying malignant process necessitating thorough clinical evaluation. Adult granulosa cell tumor of the ovary (AGCT), a subtype of ovarian sex cord-stromal tumors, is characterized by its low prevalence but generally favorable prognosis.^[1]^ Nevertheless, it is essential to recognize that approximately one-third of AGCT patients are at risk of recurrence within 4 to 7 years following diagnosis, with a significant mortality rate of up to 50% attributed to these recurrences.^[2]^ The hallmark clinical manifestations of AGCT include abnormal vaginal bleeding and the presence of a unilateral ovarian mass. Ascites, an infrequent symptom, is observed in only 18.6% to 21% of patients at initial diagnosis, while the co-occurrence of pleural effusion is exceedingly rare.^[3]^

This case report aims to elucidate the clinical presentation, diagnostic complexities, and therapeutic strategies in a patient with AGCT presenting with pleural effusion. By documenting this case, we seek to enhance the understanding of atypical manifestations of AGCTs and to provide insights into their clinical management, thereby contributing to the existing literature on this rare condition.

## 2. Case report

This case report details a postmenopausal woman in her 60s with a 20-year history of amenorrhea and no prior hormone replacement therapy. The patient initially presented with a 1-month history of persistent chest tightness and dyspnea. She sought care at a traditional Chinese medicine hospital, where chest radiography revealed right-sided pulmonary atelectasis accompanied by pleural effusion. Given the persistent symptoms, diagnostic thoracentesis was performed under ultrasound guidance, yielding 500 mL of hemorrhagic fluid. Cytological analysis of the pleural fluid revealed the presence of neutrophils, lymphocytes, and mesothelial cells, with no evidence of malignant cells. Although antibiotic treatment provided partial symptomatic relief, her respiratory symptoms persisted, and the pleural effusion showed no significant reduction. A subsequent computed tomography scan identified a pelvic mass, prompting her referral to our institution for further evaluation and management. Regrettably, due to the imaging studies being conducted at an external facility, high-resolution image data are unavailable for inclusion in this report.

The patient’s medical history was notable for poorly controlled hypertension, with no prior history of diabetes mellitus, renal disease, hepatitis, or tuberculosis. Physical examination revealed senile changes in the external genitalia, a patent vaginal canal, a smooth cervix, and an atrophic uterus. A cystic mass with restricted mobility and mild tenderness was palpated posterior to the right side of the uterus. No abnormalities were detected in the left adnexal region. Thoracoscopic evaluation demonstrated no evidence of pleural neoplasms or other pathological changes.

**Figure 1.**
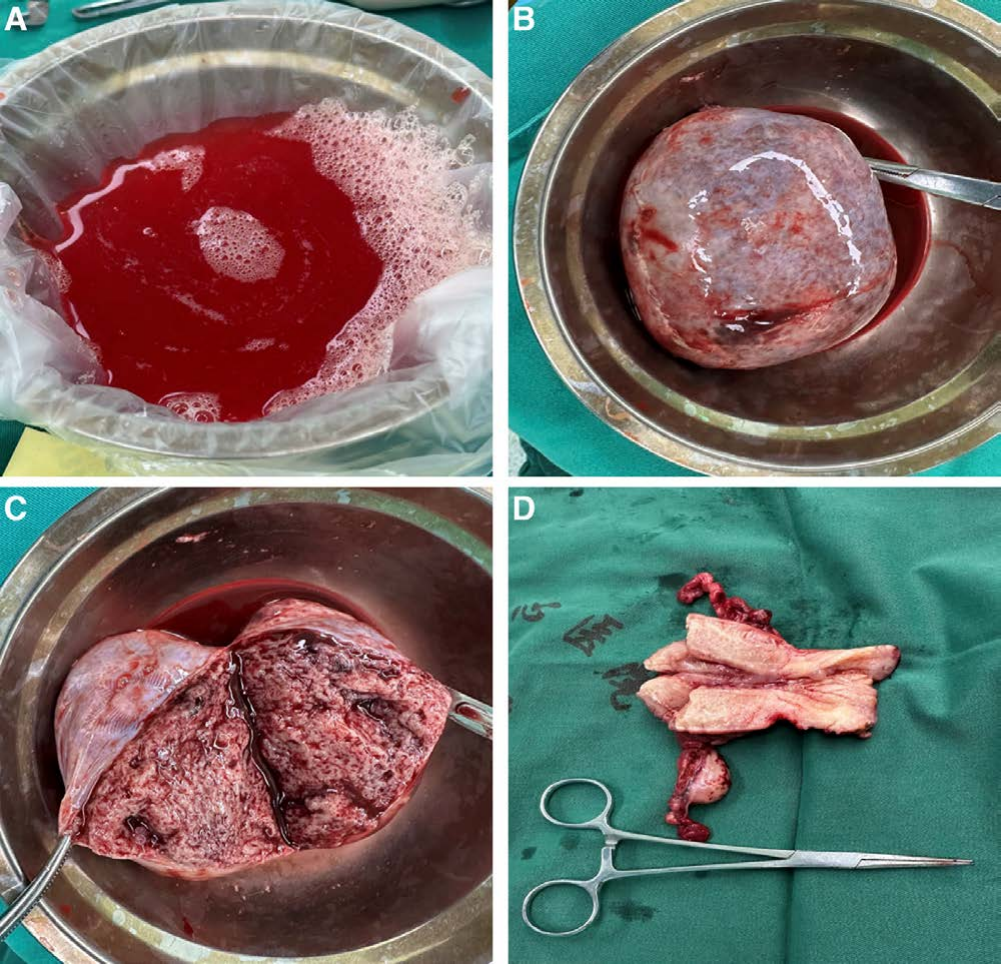
Intraoperative findings. (A) Hemorrhagic ascites; (B, C) Ovarian tumors; (D) Uterus and ovaries.

Given the complexity of the patient’s clinical presentation, an exploratory laparotomy was undertaken for both diagnostic and therapeutic purposes. Intraoperative findings revealed pale red ascites and a large tumor in the left ovarian region, measuring approximately 10 × 9.7 cm, with an intact capsule (Fig. 1A–C). Intraoperative frozen section analysis suggested a sex cord-stromal tumor. Cytological examination of the ascitic fluid identified scattered lymphocytes and segmented neutrophils against a background of abundant red blood cells, with no evidence of malignant cells. Consequently, a total hysterectomy and bilateral salpingo-oophorectomy were performed (Fig. 1D).

Postoperatively, the volume of pleural drainage fluid markedly decreased to 200 mL on the first postoperative day. By the second postoperative day, pleural drainage ceased entirely, and pelvic drainage volume was minimal, approximately 15 mL. In light of these favorable outcomes, both the pelvic and pleural drainage tubes were removed. Notably, the pleural drainage tube had been indwelling for nearly 2 weeks prior to removal. Subsequent routine histopathological examination and immunohistochemical analysis confirmed the diagnosis of an AGCT, with a Ki-67 labeling index of approximately 20%.

At the 3-month postoperative follow-up, the patient was asymptomatic, and clinical examinations revealed no evidence of disease recurrence. Specifically, there was no recurrence of pleural effusion or pelvic/abdominal fluid accumulation, indicating a successful clinical outcome.

## 3. Discussion

Meigs syndrome is classically defined by the triad of ascites, pleural effusion, and a benign ovarian fibroma.^[4]^ A hallmark of this syndrome is the spontaneous resolution of effusion symptoms following surgical removal of the tumor. In this case, the persistence of pleural effusion despite initial treatment and the identification of a pelvic mass during diagnostic evaluation raised clinical suspicion for Meigs syndrome. However, the postoperative histopathological findings confirming an AGCT, coupled with the resolution of ascites and pleural effusion after tumor resection, align more closely with a diagnosis of atypical Meigs syndrome. This case underscores the importance of including Meigs syndrome in the differential diagnosis of refractory pleural effusion, particularly when patients initially present with respiratory symptoms in pulmonary or respiratory medicine settings.^[5,6]^ For pulmonologists, unexplained pleural effusion should prompt a comprehensive differential diagnosis that encompasses a broad spectrum of conditions, including gynecological pathologies.

In the management of AGCTs, total hysterectomy and bilateral salpingo-oophorectomy are regarded as the definitive treatment. While some studies recommend against routine lymph node dissection due to the low incidence of lymph node metastasis,^[7,8]^ others highlight the importance of evaluating retroperitoneal lymph node status, particularly advocating for lymph node dissection in cases of lymph node enlargement.^[9]^ In this case, the absence of palpable lymph node enlargement and the presence of an intact tumor capsule guided the decision to perform a total hysterectomy and bilateral salpingo-oophorectomy without lymph node dissection.

The choice of surgical approach, whether laparoscopic or open, has been demonstrated to be safe, with laparoscopic surgery offering specific advantages such as reduced postoperative recovery time.^[10]^ However, laparoscopic procedures may pose risks, including changes in body position, increased intra-abdominal pressure, or pneumoperitoneum, which can exacerbate or induce pleural effusion.^[11]^ Additionally, patients with ovarian tumors accompanied by ascites and pleural effusion are at heightened risk for intraoperative oxygen desaturation due to the potential worsening of pleural effusion.^[12]^ Consequently, anesthesiologists must carefully select anesthetic techniques tailored to the patient’s specific condition. In this case, traditional open abdominal surgery was ultimately chosen to mitigate these risks and ensure optimal surgical outcomes.

This case report describes a postmenopausal woman presenting with chest tightness and dyspnea. Despite targeted treatments for respiratory conditions, her symptoms persisted, prompting further diagnostic investigations that ultimately revealed an ovarian tumor. This case highlights the critical importance of maintaining a high index of suspicion for underlying neoplasms in postmenopausal women presenting with nonspecific symptoms, underscoring the necessity for timely and comprehensive diagnostic evaluations.

The successful management of this case exemplifies the value of multidisciplinary collaboration, involving specialties such as cardiology, respiratory medicine, anesthesiology, and gynecology, and demonstrates the advantages of a comprehensive hospital setting in addressing complex clinical scenarios. Additionally, this case reinforces the importance of early surgical intervention for ovarian solid lesions, regardless of their suspected benign or malignant nature, to establish a definitive pathological diagnosis and guide subsequent therapeutic strategies.

Through a detailed analysis of the clinical presentation, diagnostic workup, and treatment approach in this case, this article aims to provide valuable insights and serve as a reference for the diagnosis and management of similar cases in clinical practice.

## 4. Conclusion

This case report outlines a comprehensive clinical evaluation, multidisciplinary collaboration, and surgical intervention that led to the diagnosis of an AGCT of the left ovary associated with atypical Meigs syndrome. The case highlights the paramount importance of early detection and prompt intervention for pelvic masses in postmenopausal women, as well as the indispensable role of multidisciplinary teamwork in addressing complex clinical presentations. Enhancing our understanding of such rare conditions and improving early diagnostic capabilities are crucial steps toward optimizing patient outcomes and advancing clinical management strategies in the future.

## Author contributions

**Conceptualization:** Minghan Chai, Qing Xue.

**Data curation:** Minghan Chai, Qiuli Jin.

**Formal analysis:** Qiuli Jin.

**Project administration:** Ying Zhao.

**Writing – original draft:** Jie Zhang.

**Writing – review & editing:** Qing Xue.

## References

[1] Alhusaini H Elshenawy MA Badran A. Adult-type ovarian granulosa cell tumour: treatment outcomes from a single-institution experience. Cureus. 2022;14:e31045. 36475202 10.7759/cureus.31045 PMC9719055

[2] Färkkilä A Haltia UM Tapper J McConechy MK Huntsman DG Heikinheimo M. Pathogenesis and treatment of adult-type granulosa cell tumor of the ovary. Ann Med. Aug. 2017;49:435–47. 10.1080/07853890.2017.1294760 28276867

[3] Li X Tian B Liu M Miao C Wang D. Adult-type granulosa cell tumor of the ovary. Am J Cancer Res. 2022;12:3495–511. 36119817 PMC9442026

[4] Brun JL. Demons syndrome revisited: a review of the literature. Gynecol Oncol. 2007;105:796–800. 17433421 10.1016/j.ygyno.2007.01.050

[5] Miyawaki E Naito T Kasamatsu Y. Pseudo-Meigs’s syndrome. BMJ Case Rep. 2021;14:e241337. 10.1136/bcr-2020-241337 PMC7868198 33541974

[6] Hou YY Peng L Zhou M. Meigs syndrome with pleural effusion as initial manifestation: A case report. World J Clin Cases. 2021;9:5972–9. 34368316 10.12998/wjcc.v9.i21.5972 PMC8316948

[7] Colombo N Peiretti M Garbi A Carinelli S Marini C Sessa C. Non-epithelial ovarian cancer: ESMO Clinical Practice Guidelines for diagnosis, treatment and follow-up. Ann Oncol. 2012;23(Suppl 7):vii20–6. 22997450 10.1093/annonc/mds223

[8] Kuru O Boyraz G Uckan H. Retroperitoneal nodal metastasis in primary adult type granulosa cell tumor of the ovary: can routine lymphadenectomy be omitted? Eur J Obstet Gynecol Reprod Biol. 2017;219:70–3. 29055817 10.1016/j.ejogrb.2017.10.010

[9] Zhao D Zhang Y Ou Z Zhang R Zheng S Li B. Characteristics and treatment results of recurrence in adult-type granulosa cell tumor of ovary. J Ovarian Res. 2020;13:19. 32059683 10.1186/s13048-020-00619-6 PMC7020364

[10] Fotopoulou C Savvatis K Braicu EI. Adult granulosa cell tumors of the ovary: tumor dissemination pattern at primary and recurrent situation, surgical outcome. Gynecol Oncol. 2010;119:285–90. 20637497 10.1016/j.ygyno.2010.06.031

[11] Kim KH Byun JW Kwon GM Shim JH. Massive pleural effusion in ovarian tumor patient during laparoscopic surgery. Korean J Anesthesiol. 2013;65(6 Suppl):S145–6. 24478852 10.4097/kjae.2013.65.6S.S145 PMC3903840

[12] Hori G Akatsu M Nemoto C Iida H Isosu T Murakawa M. A case of respiratory distress due to massive pleural effusion after surgery for ovarian tumor. Masui. 2013;62:362–4. 23544347

